# Supplementary material for: Increased reproductive tract infections among secondary school girls during the COVID-19 pandemic: associations with pandemic related stress, mental health, and domestic safety
Source: medRxiv. 2024 Feb 18:2024.02.17.24302973. Preprint. [Version 1] doi: 10.1101/2024.02.17.24302973 (PMC10889038; doi:10.1101/2024.02.17.24302973)
Supplement: Supplement 1 [file media-1.pdf]

Supplementary Material

Increased reproductive tract infections among secondary school girls during the COVID-19 pandemic: associations with pandemic related stress, mental health, and domestic safety

***Supriya D. Mehta, Debarghya Nandi, Fredrick Otieno, Garazi Zulaika, Elizabeth Nyothach, Walter Agingu, Runa Bhaumik, Linda Mason, Anna Maria van Eijk, Penelope A Phillips-Howard**

*** Correspondence:** Corresponding Author: Supriyad@uic.edu; Supriya_Mehta@rush.edu

Supplemental Table 1. Distribution of responses to COVID-19 related stress questions[1] by study time point.

Please tell us whether you agree, disagree, or don't know for the following statements.	30 Month visit, N=394 n (%)	36 Month visit, N=327 n (%)	48 Month visit, N=365 n (%)
**I am very worried about getting the coronavirus/COVID**			
Agree	306 (77.7)	259 (79.2)	272 (74.5)
Disagree	57 (14.5)	55 (16.8)	75 (20.6)
Don't know	31 (7.9)	13 (4.0)	18 (4.9)
**I am very worried about my family or friends getting the coronavirus/COVID**			
Agree	312 (79.2)	267 (81.6)	279 (76.4)
Disagree	54 (13.7)	45 (13.8)	68 (18.3)
Don't know	28 (7.1)	15 (4.6)	18 (4.9)
**I am very worried about giving someone else the coronavirus/COVID**			
Agree	286 (72.6)	234 (71.6)	256 (70.1)
Disagree	78 (19.8)	74 (22.6)	88 (24.1)
Don't know	30 (7.6)	19 (5.8)	21 (5.8)
**I have had a hard time sleeping because of the coronavirus/COVID**			
Agree	131 (33.2)	107 (32.7)	141 (38.6)
Disagree	239 (60.7)	200 (61.2)	195 (53.4)
Don't know	24 (6.1)	20 (6.1)	29 (8.0)
**I have had difficulties concentrating because of the coronavirus/COVID**			
Agree	150 (38.1)	132 (40.4)	148 (40.5)
Disagree	215 (54.6)	179 (54.7)	190 (52.1)
Don't know	29 (7.4)	16 (4.9)	27 (7.4)
**Thinking about the coronavirus/COVID makes me anxious**			
Agree	224 (56.8)	191 (58.4)	205 (56.2)
Disagree	146 (37.1)	120 (36.7)	134 (36.7)
Don't know	24 (6.1)	16 (4.9)	26 (7.1)
**I am feeling overwhelmed by the coronavirus/COVID**			
Agree	174 (44.2)	126 (38.5)	159 (43.6)
Disagree	177 (44.9)	169 (51.7)	170 (46.6)
Don't know	43 (10.9)	32 (9.8)	36 (9.9)
**I am worried about money because of the coronavirus/COVID**			
Agree	246 (62.4)	224 (68.5)	256 (70.1)
Disagree	126 (32.0)	93 (28.4)	94 (25.7)
Don't know	22 (5.6)	10 (3.1)	15 (4.1)

[1] COVID-19 related distress adapted from [25].

STROBE Statement—checklist of items that should be included in reports of observational studies

	Item No	Recommendation	Page number
**Title and abstract**	1	(*a*) Indicate the study's design with a commonly used term in the title or the abstract	Abstract
		(*b*) Provide in the abstract an informative and balanced summary of what was done and what was found	Abstract
**Introduction**			
Background/rationale	2	Explain the scientific background and rationale for the investigation being reported	Background Pages 4-5
Objectives	3	State specific objectives, including any prespecified hypotheses	Background Page 5 paragraph 4
**Methods**			
Study design	4	Present key elements of study design early in the paper	Methods Page 6 "Study Design and Participants"
Setting	5	Describe the setting, locations, and relevant dates, including periods of recruitment, exposure, follow-up, and data collection	Methods Pages 6-7
Participants	6	(*a*) *Cohort study*—Give the eligibility criteria, and the sources and methods of selection of participants. Describe methods of follow-up<br>*Case-control study*—Give the eligibility criteria, and the sources and methods of case ascertainment and control selection. Give the rationale for the choice of cases and controls<br>*Cross-sectional study*—Give the eligibility criteria, and the sources and methods of selection of participants	Methods Pages 6-7
		(*b*) *Cohort study*—For matched studies, give matching criteria and number of exposed and unexposed<br>*Case-control study*—For matched studies, give matching criteria and the number of controls per case	NA
Variables	7	Clearly define all outcomes, exposures, predictors, potential confounders, and effect modifiers. Give diagnostic criteria, if applicable	Methods Pages 8-12
Data sources/ measurement	8	For each variable of interest, give sources of data and details of methods of assessment (measurement). Describe comparability of assessment methods if there is more than one group	Methods Pages 7-10
Bias	9	Describe any efforts to address potential sources of bias	Methods Pages 10-12
Study size	10	Explain how the study size was arrived at	Methods Page 6
Quantitative variables	11	Explain how quantitative variables were handled in the analyses. If applicable, describe which groupings were chosen and why	Methods Pages 7-12
Statistical methods	12	(*a*) Describe all statistical methods, including those used to control for confounding	Methods Pages 10-12
		(*b*) Describe any methods used to examine subgroups and interactions	NA
		(*c*) Explain how missing data were addressed	Methods Page 8, 10
		(*d*) *Cohort study*—If applicable, explain how loss to follow-up was addressed	Methods Page 7-8
		(*e*) Describe any sensitivity analyses	NA
**Results**			

Participants	13	(a) Report numbers of individuals at each stage of study—eg numbers potentially eligible, examined for eligibility, confirmed eligible, included in the study, completing follow-up, and analysed	Figure 1, and throughout tables
		(b) Give reasons for non-participation at each stage	NA
		(c) Consider use of a flow diagram	Figure 1
Descriptive data	14	(a) Give characteristics of study participants (eg demographic, clinical, social) and information on exposures and potential confounders	p. 13 and Table 1
		(b) Indicate number of participants with missing data for each variable of interest	Tables 1 and 2
		(c) *Cohort study*—Summarise follow-up time (e.g., average and total amount)	Figure 1 and Table 1
Outcome data	15	*Cohort study*—Report numbers of outcome events or summary measures over time	Table 1
		Case-control study—Report numbers in each exposure category, or summary measures of exposure	NA
		Cross-sectional study—Report numbers of outcome events or summary measures	NA
Main results	16	(*a*) Give unadjusted estimates and, if applicable, confounder-adjusted estimates and their precision (eg, 95% confidence interval). Make clear which confounders were adjusted for and why they were included	Tables 3, 4, 5 summarized in pp. 13-14
		(*b*) Report category boundaries when continuous variables were categorized	NA
		(*c*) If relevant, consider translating estimates of relative risk into absolute risk for a meaningful time period	NA
Other analyses	17	Report other analyses done—eg analyses of subgroups and interactions, and sensitivity analyses	NA
**Discussion**			
Key results	18	Summarise key results with reference to study objectives	pp. 15-16
Limitations	19	Discuss limitations of the study, taking into account sources of potential bias or imprecision. Discuss both direction and magnitude of any potential bias	pp.19
Interpretation	20	Give a cautious overall interpretation of results considering objectives, limitations, multiplicity of analyses, results from similar studies, and other relevant evidence	pp. 15-19
Generalisability	21	Discuss the generalisability (external validity) of the study results	pp.19
**Other information**			
Funding	22	Give the source of funding and the role of the funders for the present study and, if applicable, for the original study on which the present article is based	Title page
